# Comparison of Policy‐Relevant Air Quality Metrics Calculated With Sparse In Situ Monitoring and Contiguous Satellite‐Derived Data

**DOI:** 10.1029/2025GH001586

**Published:** 2026-07-07

**Authors:** Summer Acker, Tracey Holloway, Kevin M. Stewart, Aaron van Donkelaar, Randall V. Martin

**Affiliations:** ^1^ Nelson Institute Center for Sustainability and the Global Environment University of Wisconsin—Madison Madison WI USA; ^2^ Department of Atmospheric and Oceanic Sciences University of Wisconsin—Madison Madison WI USA; ^3^ American Lung Association Chicago IL USA; ^4^ Department of Energy, Environmental & Chemical Engineering Washington University in St. Louis St. Louis MO USA

**Keywords:** satellite‐derived PM_2.5_, monitor‐satellite alignment, risk assessment, NAAQS

## Abstract

Compliance with the U.S. National Ambient Air Quality Standards (NAAQS) traditionally relies on a sparse network of ground‐based monitors. We compare two methods for assessing whether U.S. counties are above or below the annual fine particulate matter (PM_2.5_) NAAQS of 9.0 μg/m^3^: the monitor‐based methodology used to calculate county design values (CDVs; an EPA metric representing the maximum, in‐county PM_2.5_ monitor value over a 3‐year annual average), and a satellite‐based methodology that computes county design value equivalents (CDVEs) from Washington University's global satellite‐derived PM_2.5_ data product. The satellite‐based approach uses the 90th percentile PM_2.5_ grid value in a county and shows strong overall agreement with monitor‐based CDVs across 536 monitored U.S. counties (*r* = 0.76; *r*_s_ = 0.74). Counties were classified as aligned positives (APs), aligned negatives (ANs), non‐aligned positives (NPs), or non‐aligned negatives (NNs) based on whether CDVEs align or don't align with CDVs on NAAQS status. Risk factors likely contributing to larger differences between CDVEs and CDVs within non‐aligned counties include ≤2 monitors, low (<7 μg/m^3^) or high (>10 μg/m^3^) CDVs, low (<0.05%) or high (>0.1%) monitor coverage, large county size, wildfires, mountains, deserts, and low urbanization. In non‐aligned counties, differences grow with the number of risk factors, with seven‐risk‐factor counties showing 5x higher median differences than those with one. High error‐risk counties cluster in the Western U.S.; low error‐risk counties are in the Midwest and East. These findings highlight trade‐offs between sparse and contiguous data for regulatory assessments and support integration of satellite‐derived data into policy frameworks.

## Plain Language Summary

The majority of U.S. counties lack ground‐based monitors to track health‐damaging fine particulate matter (PM_2.5_). Satellite data may be used to calculate PM_2.5_, but satellite‐derived values can differ from ground monitors. We compared county‐level satellite‐derived PM_2.5_ with county‐level EPA monitor data across the U.S. to understand where and why these two data sets agree or disagree. We found that large counties with wildfires, mountains, deserts, few monitors, and either low or high pollution levels were more likely to show mismatches. These differences were most common in the western U.S. and grew larger as more risk factors were present. These findings highlight where caution is needed when using both satellite and monitor data for air quality management and policy.

## Key Points

- Monitors and satellite‐derived data align in showing annual PM_2.5_ is below 9.0 μg/m^3^ in 70% and above in 12% of monitored U.S. counties
- In 18% of counties, monitors and satellite‐derived data are not aligned; satellite data are higher than monitors in 46% and lower in 54%
- Likely drivers of satellite‐monitor differences include wildfires, pollution level, county size, monitor coverage, mountains, and deserts

## 1 Introduction

Air quality management in the United States (U.S.) relies on regulatory monitors to assess pollution levels and inform compliance with the Environmental Protection Agency's (EPA) National Ambient Air Quality Standards (NAAQS). These monitors are primarily managed by U.S. states, tribes, and local agencies, in compliance with EPA guidelines for quality control and quality assurance. While often considered the “gold standard” for regulatory application, these monitors have limited spatial coverage, with over 80% of U.S. counties lacking a fine particulate matter (PM_2.5_) monitor (Holloway et al., 2025).

Past work from our group suggested that spatially contiguous satellite‐derived data for PM_2.5_ could offer an alternate approach to evaluate county‐scale pollution levels, and comparison with the annual PM_2.5_ NAAQS (Holloway et al., 2026). Although the comparison of satellite‐derived data to the NAAQS bears relevance to regulatory compliance (Sullivan & Krupnick, 2019), we approach this question primarily in terms of relevance to the American Lung Association's (ALA) public outreach work through the annual State of the Air (SOTA) report. The ALA SOTA ranks U.S. counties based on annual PM_2.5_ concentrations using EPA‐calculated county design values (CDVs), which are calculated from ground‐based monitors. The SOTA determines if a U.S. county is “passing” or “failing” the PM_2.5_ NAAQS, acting as an air quality “report card,” with the ALA’s most recent reports using the 2024 NAAQS standard of 9.0 μg/m^3^ as the pass/fail threshold (American Lung Association, 2025b). Here we extend our previous analysis (Holloway et al., 2026) to characterize the factors contributing to alignment or non‐alignment between monitor‐based and satellite‐derived county‐level above/below the 2024 NAAQS threshold classifications, with emphasis on the implications of using sparse versus contiguous data sources.

The implications of calculating summary metrics with sparse versus contiguous data have been considered across many disciplines, but a few examples include public health, conservation biology, meteorology, and air quality. In public health, sparse clinical survey data (e.g., small area estimates or SAEs) has been compared to approximately contiguous electronic health records (EHRs), with EHRs having broader spatial and temporal coverage, but less reliability as they overestimated health outcomes compared to SAEs (Nielsen et al., 2024). In conservation biology, sparse species observation data can suffer from under‐coverage and sampling bias, while species distribution models offer spatial completeness but depend on assumptions that may reduce transparency and accuracy for decision‐making (Sofaer et al., 2019). In meteorology, rainfall gauges (sparse) remain the most accurate measurements, but satellite precipitation products significantly improved spatial coverage, though their performance varied with elevation and often underestimated or overestimated precipitation depending on conditions (Kumar et al., 2022). In air quality, a study developed daily PM_2.5_ estimates at the county, ZIP code, and census tract levels across 11 western U.S. states using satellite aerosol optical depth (AOD) and other predictors, finding strong agreement with monitors (*r* ≈ 0.70) but reduced performance in less urban areas (Reid et al., 2021). EPA monitors often underrepresent intra‐county variability, and with empirical model‐based surface PM_2.5_, it was found that 44% of U.S. urban areas in unmonitored census tracts were failing the 2024 NAAQS (Wang et al., 2024). About 6.73 million people in 2020 were living in counties in attainment of the PM_2.5_ NAAQS, but were in census tracts that were above the NAAQS threshold (Dobkin & Kerr, 2025).

The calculation of regulatory metrics for air quality bears some unique data characteristics that stand out from prior studies. Air quality is characterized at the county scale for ALA applications (American Lung Association, 2024, 2025a, 2025b), NAAQS attainment status (also at the partial‐county; Cropper et al., 2025; US EPA, 2025a), and using the US EPA's CO‐Benefits Risk Assessment (COBRA) screening tool for county‐level air quality health benefits (Mailloux et al., 2022) or air quality and environmental justice impacts from decarbonization (Gallagher & Holloway, 2022). However, the placement of air monitors does not follow a standard sampling methodology. Rather, monitors may be placed to capture the highest expected pollutant concentrations or typical levels in populated areas, to evaluate emissions from major sources, or to track regional pollution transport (EPA, 2008). This targeted approach does not aim to provide spatial representativeness, and in fact it may be mischaracterizing pollution levels—particularly in areas where pollution is highly variable (e.g., urban areas), in complex terrain such as mountains (Giovannini et al., 2020), or where few monitors exist (Snyder et al., 2013).

Both the rationale for monitor siting and the number of monitors per unit area vary widely across the U.S., as do county sizes (Figure S1 in Supporting Information S1; Holloway et al., 2026). Most monitored U.S. counties have just one or two monitors, and many large counties—particularly in the western U.S.—cover vast geographic areas that encompass highly heterogeneous pollution sources and population distributions. This creates challenges when using sparse networks to characterize county‐ or sub‐county level attainment status. To address such challenges, EPA and state agencies conduct ambient air monitoring network assessments every 5 years, as mandated by 40 CFR 58.10(e), to evaluate whether existing networks meet their monitoring objectives and to recommend adjustments where needed (US EPA, 2007). The purpose of these network assessments is to optimize monitoring resources to protect public and environmental health while supporting scientific and regulatory needs. Assessments consider whether existing sites remain necessary, whether new sites are needed in underserved areas, and whether emerging technologies could improve monitoring capabilities. These evaluations also account for changes in population distribution, emission patterns, and air quality trends, ensuring the network remains responsive to evolving air quality challenges and long‐term public health goals. However, these assessments are limited to evaluating areas with existing monitors and do not systematically address gaps in unmonitored regions. This focus, combined with the network's uneven spatial distribution, varying county sizes, and differences in monitor density across regions, reveals critical shortcomings in capturing localized air pollution variability and population exposures.

In our previous analysis (Holloway et al., 2026), data from each monitor is used to calculate a design value (DV), where the DV algorithm is specified in the NAAQS for each regulated pollutant. For annual PM_2.5_, the DV is calculated by the EPA as the four‐quarter average of quarterly averaged measurements of PM_2.5_ measured at monitors and averaged over the preceding 3 years (e.g., 2021, 2022, and 2023 are averaged together for the 2023 DV). To extend these values to the county level, the maximum of all of the monitor(s) annual average DV value(s) in a county is selected. As such, county‐level DVs (CDVs) are sensitive to the maximum monitor's location, potentially underestimating overall exposure in highly polluted areas or overestimating exposure in highly localized pollution hotspots.

Here we focus on satellite‐derived data from the Washington University in St. Louis (WashU) Atmospheric Composition Analysis Group (Hammer et al., 2023; Van Donkelaar et al., 2021). Past studies have compared satellite‐derived PM_2.5_ with ground measurements, typically comparing values from a single grid cell to monitor values (Bai et al., 2022; Van Donkelaar et al., 2010), or averaging both measurements and grid values over an area (Vaidyanathan et al., 2013). Spatially contiguous data sets offer the potential to detect hotspots and gradients which can help to identify communities experiencing higher levels of pollution that may be unmonitored (Gardner‐Frolick et al., 2022). In regions with complex terrain (e.g., mountain ranges), where spatial variability in meteorological and air quality conditions is high, spatially contiguous remote sensing technologies are especially useful (Giovannini et al., 2020). Contiguous data sources enable broader surveillance and help fill gaps between monitors, but they also carry limitations related to temporal resolution, satellite retrieval constraints (e.g., the absence of data due to cloud cover or night time), data uncertainty, modeling assumptions, and the high computational cost of modeling (Gardner‐Frolick et al., 2022; Holloway et al., 2025; Potts et al., 2024). The challenge, therefore, is determining how to fairly and effectively translate contiguous data into policy‐relevant frameworks traditionally built on point‐based observations.

For comparison with CDVs, Holloway et al. (2026) found that the 90th percentile of satellite‐derived data grid cells within each county, or the county‐design value equivalent (CDVE), yielded superior agreement with EPA CDVs (*r* = 0.76; *r*_s_ = 0.74). While the overall CDV versus CDVE calculation speaks to the agreement of the two methods, the decision‐relevance of this analysis relates to the alignment of the county‐scale values with the annual PM_2.5_ NAAQS threshold. We consider here the 2024 PM_2.5_ NAAQS of 9.0 μg/m^3^. CDVEs are compared against CDVs, with “alignment” defined as CDVEs agreeing with CDVs on whether a county exceeds the NAAQS threshold, and “non‐alignment” defined as CDVEs and CDVs not agreeing on whether there's an exceedance.

Previous studies have focused on evaluating the spatial representativeness of the monitoring network itself, quantifying how well each site captures surrounding air quality conditions (e.g., Bai et al., 2022, 2025). Our goal is not to assess the representativeness of individual monitors, but to evaluate how well satellite‐derived PM_2.5_ data reproduce county‐scale metrics that are directly comparable to those used in regulatory applications (CDVs). The specific focus of this analysis is where these county scale metrics differ.

Here we identify and evaluate the characteristics of counties that lead to differences between CDVEs and CDVs in counties where CDVEs and CDVs don't align on whether a county is above or below the NAAQS and where they do align. An improved understanding of disagreement between CDV and CDVE characteristics serves multiple goals. First, it supports improved methods to characterize NAAQS attainment from novel data sources, especially satellite‐based data fusion methods. Second, such a characterization supports uncertainty characterization of NAAQS designations, including sensitivity to monitor number and placement for CDV assessments, and factors affecting uncertainty in CDVE assessments. Finally, an improved understanding of monitor network design for CDV characterization may inform monitor placement approaches. This study is intended to inform public health and outreach applications that present county‐scale PM_2.5_ data informed by the CDV. By improving understanding of CDVE–CDV differences, these applications may consider expanding spatial coverage of county‐scale health metrics to cover the entire U.S. We evaluate several explanatory variables including monitor density, county area, monitor coverage, physical features (viz., mountains and deserts), pollution level, urbanization, satellite‐derived data uncertainty, and the presence of wildfire activity. The remainder of this paper is structured as follows: Section 2 describes the data and methods; Section 3 presents results, beginning with county classifications based on CDV and CDVE agreement, identification of “risk factors” driving differences, and a risk assessment to classify monitored and unmonitored counties by error risk; and Section 4 discusses the implications for air quality assessments using sparse versus contiguous data.

## 2 Methods

Here we present the data sets used (Section 2.1), the process for calculating CDVs and CDVEs (Section 2.2), the methodology and reasoning for county classification (Section 2.3), the set of explanatory variables used to interpret these differences (Section 2.4), and the risk assessment methodology used to identify counties that are likely to exhibit greater differences between CDVs and CDVEs (Section 2.5).

### 2.1 Monitor Data

We use monitor‐based county‐level design values (CDVs) calculated by the EPA for 2021 through 2023 for all U.S. states (US EPA, 2016). We analyze CDVs for 536 of the EPA monitored U.S. counties. Following the approach of the ALA SOTA (American Lung Association, 2025b), we use CDVs to determine if a county is below (≤9.0 μg/m^3^) or above (>9.0 μg/m^3^) the EPA PM_2.5_ 2024 NAAQS standard.

### 2.2 Satellite‐Derived Data

From previous analysis (Holloway et al., 2026), we determined that the Global/Regional (GL) Estimates V5.GL.05 satellite‐derived PM_2.5_ data fusion product (Hammer et al., 2023; Van Donkelaar et al., 2021) from the Atmospheric Composition Analysis Group at WashU, is the best satellite‐derived data set currently available for use in grading counties by PM_2.5_. The satellite‐derived GL data set integrates observations from multiple satellite platforms (e.g., MODIS, MISR, SeaWiFS, VIIRS) using several AOD retrieval algorithms and converts them to surface‐level PM_2.5_ concentrations using the GEOS‐Chem chemical transport model, followed by statistical fusion with ground‐based PM_2.5_ monitoring data for improved accuracy (Van Donkelaar et al., 2021). The data is available from 1998 through 2023 on a 0.01° × 0.01° grid which covers all of the contiguous U.S. and all of Hawaii and nearly all of Alaska (except for North Slope Borough). Filtering was applied to each of the annual average data sets (2021, 2022, and 2023) using the satellite‐derived uncertainty estimate file that corresponds to each year of data. We removed all grid cells from each year that had an uncertainty greater than 90%. We then calculated a 3‐year average of the satellite‐derived GL 2021–2023 filtered annual average PM_2.5_ data and then found the 90th percentile of all of the remaining grids in a county to calculate the county design value equivalents (CDVEs). The 90th percentile was determined to be the best percentile for comparison with CDVs, as it overall had the best agreement (for both Pearson correlation and Spearman Rank coefficient) out of all tested percentiles (0.1–0.99) among the DV periods studied in Holloway et al. (2026). The 2020 U.S. County shapefile from the U.S. Census Bureau Cartographic Boundary Files (US Census Bureau, 2025) was used to allocate grid cells to a county. This analysis focuses on the 536 EPA monitored counties, although previous analysis analyzed both EPA monitored and unmonitored counties using the satellite‐derived GL data (Holloway et al., 2026). See Holloway et al. (2026) for additional details on the calculation of CDVs and CDVEs as well as the calculation and discussion of the correlations between CDVs and CDVEs across DV periods.

### 2.3 Classification

Previous analysis (Holloway et al., 2026) found that only 63 counties were identified by both CDVs and CDVEs as having PM_2.5_ concentrations greater than the 2024 NAAQS, while CDVs found 115 counties and CDVEs found 108 counties. To explore sources of disagreement, we define the monitors as the “ground truth” and the satellite‐derived data as the “predictor,” classifying EPA‐monitored counties into four classifications: aligned positive counties (AP; both CDV and CDVE >9 μg/m^3^ 2024 threshold; *N* = 63), aligned negative counties (AN; both ≤9 μg/m^3^; *N* = 376), non‐aligned positive counties (NP; CDV ≤9.0 μg/m^3^, CDVE >9.0 μg/m^3^; *N* = 45), and non‐aligned negative counties (NN; CDV >9.0 μg/m^3^, CDVE ≤9.0 μg/m^3^; *N* = 52). In statistical terms, these correspond to true positives, true negatives, false positives, and false negatives, respectively, following the true versus false classification methodology that derives from Stewart (2000). However, we refrain from using the terms “true” and “false” within our classifications since point‐based monitors cannot truly capture the full gradient of air pollution throughout an entire county, even though they are recognized as the more reliable and accurate instrument. There were 63 AP counties identified, 376 AN counties, 45 NP counties, and 52 NN counties.

We also conducted a sensitivity analysis to assess how small changes in CDVs and CDVEs could alter county classifications. We applied perturbations of ±0.5 μg/m^3^ to CDV and CDVE values independently and in combination, resulting in nine scenarios per county. A perturbation of this magnitude represents a realistic small shift in annual PM_2.5_ based on national variability. Using EPA's national trend data for 2000–2023 (US EPA, 2025b), we calculated that the average absolute year‐to‐year difference was 0.52 μg/m^3^ per year for the national mean annual PM_2.5_ concentration and 0.83 μg/m^3^ per year for the national 90th percentile PM_2.5_ concentration. These values indicate that ±0.5 μg/m^3^ is within the range of typical interannual variability observed in U.S. PM_2.5_ levels. These perturbations included cases where both CDV and CDVE increased by 0.5 μg/m^3^, both decreased by 0.5 μg/m^3^, one increased or decreased while the other remained fixed, or one increased or decreased while the other did the opposite. For each perturbation scenario, we evaluated counties in all initial classifications (AP, AN, NP, NN) and counted how often they were reclassified into a different category relative to the 9.0 μg/m^3^ threshold. The results of this analysis are presented in Section 3.1.

### 2.4 County‐Level Explanatory Variables and Spatial Analysis

To investigate potential causes of misalignment between monitor‐based and satellite‐derived county‐level PM_2.5_ design values, we calculated a set of explanatory variables that characterize physical, environmental, and observational aspects of each county. All variables were derived using spatial operations applied to a shared county‐level PM_2.5_ data set containing county geometries and the corresponding CDVs and CDVEs. Spatial joins were performed between this data set and external spatial data sets representing each variable of interest, as detailed in the following subsections.

Categorical variables (e.g., mountain vs. non‐mountain, desert vs. non‐desert) are compared directly across their predefined groups. For continuous variables (e.g., monitor coverage, county area, or peak monitor‐to‐grid distance), values were binned into qualitative categories (e.g., low, medium, high) based on their distributions and prior scientific knowledge, allowing for comparison of mean absolute CDVE–CDV differences across groups, consistent with the categorical variables.

#### 2.4.1 County Area

Larger counties tend to have greater spatial heterogeneity in emissions, land cover, and topography, increasing the likelihood that a few monitors will capture different pollution patterns than the satellite‐derived data. To calculate the area of each county, we reprojected the county‐level PM_2.5_ data set to an equal‐area coordinate reference system (CRS) to ensure accurate areal measurements. This was done any time an area or a distance was calculated in this study. We then computed the area of each county geometry in square kilometers.

#### 2.4.2 Wildfire Presence

Wildfires represent episodic events that can occur far from existing monitoring sites, meaning monitors may or may not capture elevated PM_2.5_ associated with these events even though they are clearly detected in satellite observations; we include wildfire influence to examine whether such extreme, spatially variable events contribute to 3‐year DV period CDV‐CDVE misalignment. We downloaded the Monitoring Trends in Burn Severity (MTBS) Burned Areas Boundaries data set (MTBS, 2025), which includes perimeters of wildfires greater than 500 acres (eastern U.S.) or 1,000 acres (western U.S.) that occurred from 1984 through 2024. We filtered this data set to include only wildfires occurring within our design value (DV) period (2021–2023). A county was classified as a “fire region” if any wildfire polygon was within or intersected the county boundary. Of the 536 monitored counties, 139 had a wildfire within the DV period, while 397 did not.

#### 2.4.3 Mountain Ranges and Deserts

We obtained the physical label areas from Natural Earth (Natural Earth, 2009), which map major physical features in the U.S. at a 10 m scale, with cartographic accuracy to the 50 m scale. This data set was filtered to retain only mountain range and desert geometries. These surface types are thought to exacerbate satellite‐ground PM_2.5_ discrepancies through mechanisms such as complex topography, where the PM_2.5_ to AOD correlation degrades due to higher terrain (He et al., 2021), or elevated surface reflectance, which degrades aerosol retrieval over bright/arid surfaces (Van Donkelaar et al., 2006). A county was labeled a “mountain region” if any mountain range intersected it, and a “desert region” if any desert geometry intersected it. Based on this classification, 237 counties intersected a mountain range, 299 did not; 14 counties intersected a desert, while 522 did not.

#### 2.4.4 Urbanization

Differences in land use, emissions, and number of monitors between urban and rural areas may affect overall alignment between monitors and satellite‐derived data. We used the 2020 U.S. Census Bureau Urban Areas TIGER/Line shapefile (U.S. Census Bureau, 2025), which defines urban areas as locations with at least 2,000 housing units or a population of at least 5,000. We performed a geometric intersection between urban area geometries and each county to obtain only the portion of each urban area that fell within each county, since there were many urban areas that intersected several counties. We then computed the percentage of county land area covered by urban areas. To distinguish significantly urbanized counties since most counties had an urban area (whether small or large), we applied a threshold: counties with ≥50% of their land area designated as urban were classified as “urban,” while those with <50% were classified as “non‐urban.” This resulted in 72 urban and 464 non‐urban counties.

#### 2.4.5 Monitor Count

Counties with few monitors may have limited spatial coverage, potentially making the CDV far less representative of the county's PM_2.5_ levels than the satellite‐derived CDVE. Using the EPA's 2023 Design Value data set (US EPA, 2016) (the same source used for CDV calculation), we extracted the locations of individual monitors with valid 2023 DVs. We counted the number of such monitors within each county to determine the monitor count per county.

#### 2.4.6 Monitor Coverage

Low or high monitor coverage may bias how well the monitoring network captures spatial gradients—either under‐representing populated regions or over‐representing localized sources relative to satellite coverage. We assessed the percentage of satellite‐derived grid cells that had monitors within them by spatially joining the individual 2023 DV monitors with the satellite‐derived PM_2.5_ grid cells. We then calculated, for each county, the percentage of satellite‐derived grid cells that contained at least one monitor. While methods such as the Concentration Similarity Frequency approach (Bai et al., 2025) can directly quantify the spatial representativeness of individual monitors using daily data, our analysis is focused on annual, county‐level metrics. Consistent with EPA's spatial representativeness designations in 40 CFR Part 58 (US EPA, 2024), which define neighborhood‐scale monitors as representative of approximately 0.5–4 km, we use the percentage of 1 km satellite‐derived grid cells per county containing at least one monitor as a proxy for the county‐scale representativeness of monitor placement.

#### 2.4.7 Grid Cell Uncertainty

During satellite‐derived data preprocessing, we removed satellite‐derived grid cells with a percent uncertainty greater than 90%. We tracked both the number and location of these removed grid cells. Using a spatial join with the county geometries, we computed the number of high‐uncertainty cells removed per county. Additionally, for all grid cells (retained and removed), we retained the percent uncertainty values to assess whether grid cells in a county tended to approach the 90% uncertainty threshold, even if they were not excluded. The number of satellite‐derived grid cells removed due to high uncertainty may affect CDVE–CDV alignment, as extensive data removal can reduce the spatial representativeness of the satellite‐derived annual averages and may bias the resulting county‐level estimates.

#### 2.4.8 Peak‐Monitor to Peak‐Grid Distance

To determine if the satellite‐derived data and the monitors were identifying the same hotspot location, we identified the highest individual monitor DV and the highest satellite‐derived grid cell value within each county. We then calculated the straight‐line (Euclidean) distance between these two peak values using projected coordinates, which approximate Earth's surface as a plane. This “peak‐monitor to peak‐grid” distance quantifies the spatial offset between the monitor‐recorded and satellite‐identified pollution maxima. If the distance between the peak monitor and peak grid cell is large, then that may contribute to greater differences between the CDVs and CDVEs.

### 2.5 Error‐Risk Assessment

To evaluate the likelihood of large differences between satellite‐derived CDVEs and monitor‐based CDVs, we developed an error‐risk scoring framework based on the explanatory variables or “risk factors” that we identify in Section 3.2.

We first determined whether each explanatory variable contributed to higher differences by comparing non‐aligned counties (NP and NN) that exhibited the variable to those that did not. A variable was classified as a “risk factor” if non‐aligned counties with the factor present had a higher mean absolute difference ($\bar{\left|\text{CDVE}-\text{CDV}\right|}$) than non‐aligned counties without it. For example, counties within wildfire regions exhibited a 66% greater mean absolute difference than non‐wildfire counties, leading us to designate wildfire regions as a high‐risk characteristic.

To statistically assess the significance of each risk factor, we applied the Mann‐Whitney U test (Mann & Whitney, 1947) to compare the distributions of absolute mean differences between non‐aligned counties (NP and NN) that exhibited a given variable and those that did not. The test was implemented using the mannwhitneyu function from the SciPy Python package (Virtanen et al., 2020). Since multiple comparisons were performed across the set of explanatory variables, we applied a Bonferroni correction (Faraway, 2016) to lessen the chance of false discoveries. In this correction, the standard significance threshold (*α* = 0.05) is divided by the number of comparisons made—in our case, ten (including two inconclusive variables)—resulting in an adjusted α level of 0.005. Variables that remained significant under this corrected threshold were considered the most robust explanatory factors contributing to CDVE–CDV differences. We found that mean absolute differences tended to increase as p‐values decreased, indicating that variables producing the largest discrepancies were also the most statistically significant; therefore, we used the mean absolute differences as the weighting metric in the error‐risk assessment.

Weights for each risk factor were computed to reflect their relative contributions to differences (Equation 1). For each explanatory variable, we calculated: (a) The mean absolute CDVE–CDV difference within non‐aligned counties for both the high‐risk and low‐risk groups across all EPA monitored counties; (b) the difference in mean absolute values between these groups; and (c) the normalized differences between the high‐risk and low‐risk groups across all risk factors such that the sum of weights equals 1. This weighting scheme assigns higher weights to factors associated with larger increases in CDVE–CDV differences.

(1) $$w_{j}=\frac{\left|{\bar{\left|\text{CDVE}-\text{CDV}\right|}}_{\text{high}-\text{risk},j}-{\bar{\left|\text{CDVE}-\text{CDV}\right|}}_{\text{low}-\text{risk},j}\right|}{\sum_{k=1}^{n}\left|{\bar{\left|\text{CDVE}-\text{CDV}\right|}}_{\text{high}-\text{risk},k}-{\bar{\left|\text{CDVE}-\text{CDV}\right|}}_{\text{low}-\text{risk},k}\right|}$$

In Equation 1, $w_{j}$ is the normalized weight for risk factor j, and n is the total number of risk factors considered.

To compute error‐risk scores for each county (Equation 2), we defined a binary indicator for each risk factor (e.g., monitor count, mountains), equal to one if the county exhibits the high‐risk characteristic and 0 otherwise. Each risk factor's binary value was multiplied by its normalized weight, and these products were summed to produce a composite error‐risk score for each county.

(2) $$R_{i}=\sum_{j=1}^{n}w_{j}\cdot I_{ij}$$

In Equation 2, $R_{i}$ is the error‐risk score for county i, $w_{j}$ is the weight for risk factor j, and $I_{ij}$ is an indicator function equal to one if county i exhibits risk factor j and 0 otherwise.

For EPA‐monitored counties, scores were calculated using all eight identified risk factors, which included monitor‐related variables (e.g., monitor count, monitor coverage percentage, and county design value bin). For unmonitored counties, only the five risk factors unrelated to monitors were used, as monitor data are not available. The weights from the monitored counties with those five risk factors were used for calculating the risk scores for unmonitored counties.

Finally, counties were categorized based on their error‐risk scores where low error‐risk corresponds to scores between 0 and 0.5, medium error‐risk corresponds to scores between 0.5 and 0.75, and high error‐risk corresponds to scores between 0.75 and 1. This approach enables the identification of counties where differences between CDVEs and CDVs are more likely to be higher due to geographic and monitor‐related risk factors.

## 3 Results

Our results reveal key spatial and contextual patterns underlying alignment and non‐alignment between monitor‐based CDVs and satellite‐derived CDVEs. First, we analyze the county classifications (Section 3.1), then identify characteristics deemed “risk factors” that explain the differences between CDVs and CDVEs (Section 3.2), and finally we conduct a risk assessment using the defined risk factors to identify low, medium, and high error‐risk counties in both EPA monitored and unmonitored counties (Section 3.3).

### 3.1 County Classifications

This section evaluates several characteristics of the four classifications (AP, AN, NP, NN) including statistical comparisons of CDV and CDVE, how many counties are within high proximity to the NAAQS threshold, the spatial distribution of classifications, and the differences between CDVEs and CDVs.

**Figure 1:**
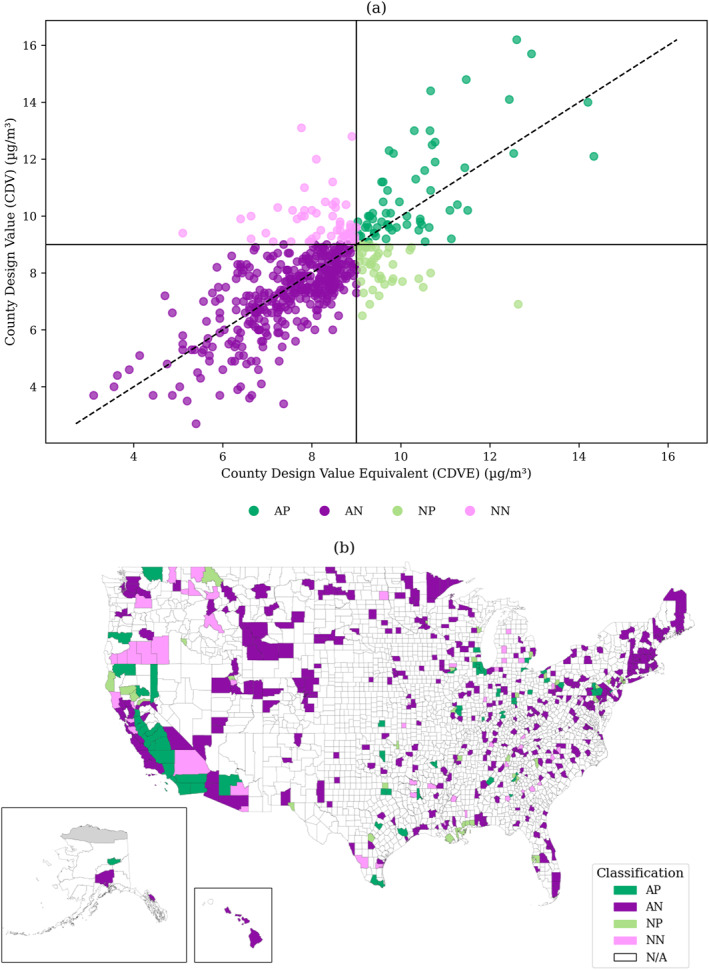
(a) The 2023 DV period U.S. CDVEs versus CDVs classified as AP (green), AN (purple), NP (light green), and NN (light pink). The horizontal and vertical lines represent the 9.0 μg/m^3^ threshold for CDVs and CDVEs and the dashed line represents the 1:1 line. (b) The spatial distribution of AP, AN, NP, and NN counties in the U.S. for the 2023 DV period. The counties shown in white are counties not containing EPA monitors and the county in gray in Alaska is a county without satellite‐derived GL data. Darker colors correspond to counties that agree, and lighter colors correspond to counties that disagree.

Figure 1 presents the classification outcomes for the 2023 DV period in both scatter and map form. Figure 1a shows a Taylor‐Russell‐style diagram, plotting CDVEs (prediction) against CDVs (ground “truth”). Vertical and horizontal lines mark the 2024 NAAQS threshold of 9.0 μg/m^3^, dividing the space into four quadrants corresponding to the classification outcomes: AP (green), AN (purple), NP (light green), and NN (light pink). Aligned counties are in darker colors (AP and AN) and non‐aligned counties (NP and NN) are in lighter colors. Most counties align near the 1:1 line indicating overall agreement between CDVEs and CDVs, which was found previously (*r* = 0.76; *r*_s_ = 0.74; Holloway et al., 2026). The density of all classifications increases near the threshold, where small differences in values lead to divergent outcomes in classification. As expected, AP counties had both CDVE and CDV means well above the threshold (10.28 and 10.76 μg/m^3^, respectively), while AN counties had lower mean values for both CDVE (7.47 μg/m^3^) and CDV (7.22 μg/m^3^). NP counties had a mean CDVE of 9.56 μg/m^3^ (range: 9.03–12.63), just above the NAAQS threshold, while their corresponding CDVs averaged 8.21 μg/m^3^ (range: 6.5–9.0), placing them just below the NAAQS threshold. NN counties had a mean CDVE of 8.18 μg/m^3^ (range: 5.1–9.0), slightly underestimating PM_2.5_ compared to their CDVs, which averaged 9.86 μg/m^3^ (range: 9.1–13.1). These patterns confirm that most misalignments between CDVEs and CDVs occur near the threshold and are typically small in absolute magnitude but are still consequential for classification outcomes.

We find that county classifications are sensitive to small perturbations of 0.5 μg/m^3^ in CDV and CDVE values, with a substantial portion of counties shifting classifications under slight increases or decreases in PM_2.5_, especially within non‐aligned counties. AP counties were relatively stable, shifting classification 85 times (15.0% of 567 perturbations), most often to NNs or NPs. AN counties exhibited the greatest stability, shifting classification in 274 cases (8.1% of 3,384 perturbations), typically reclassifying as NPs or, less commonly, as NNs or APs. In contrast, NP counties shifted classification 127 times (31.4% of 405 perturbations), primarily becoming ANs or APs. NN counties shifted 133 times (28.4% of 468 perturbations), most often reclassifying as APs or ANs. These results highlight that alignment and misalignment between satellite‐derived and monitor‐based classifications can be highly sensitive to even minor changes in PM_2.5_ concentrations, particularly in non‐aligned counties near the 9.0 μg/m^3^ threshold. However, this sensitivity likely varies across time periods; during more heavily polluted years, when more counties exceed the standard, classification outcomes may be less affected by small concentration changes.

Figure 1b illustrates the spatial distribution of classification outcomes across U.S. counties for the 2023 DV period. NNs are most common in California, the Pacific Northwest, the southern U.S., and parts of the Midwest including Michigan. NPs are concentrated in the eastern U.S., particularly in the Southeast and Mid‐Atlantic, with additional scattered counties in the Northwest. These spatial patterns of non‐alignment may reflect either limitations in satellite retrieval accuracy or differences in how well individual monitors represent county‐wide conditions, depending on local terrain, emissions, and monitor placement. The following section examines these factors in more detail. APs are heavily clustered in California's Central Valley and the Northwest, with a few appearing in the Midwest, the South, and the New York City metropolitan area. ANs are widespread and the most spatially extensive group, appearing throughout the Midwest, South, and Northeast. ANs are the only classification group with substantial presence in both the Mountain West and northeastern U.S., highlighting regions where satellite and monitor data consistently agree that counties meet the annual PM_2.5_ standard. These spatial trends point to geographic influences on classification alignment, which are further explored in Section 3.2.

The differences between CDVs and CDVEs broken down by classification are depicted in a histogram in Figure S1 in Supporting Information S1. Negative differences indicate monitor‐based CDVs are recording the highest pollution and positive differences indicate satellite‐derived CDVEs recording the highest pollution, with values closer to zero indication better agreement between monitors and satellite‐derived data. AP county differences (Figure S1a in Supporting Information S1) range from −3.73 to 2.23 μg/m^3^ with a mean difference of −0.48 μg/m^3^, indicating that AP counties are more likely to have higher CDVs than CDVEs. AN county differences (Figure S1b in Supporting Information S1) range from 0.93 to 3.97 μg/m^3^ with a mean difference of 0.24 μg/m^3^, indicating that AN counties are more likely to have higher CDVEs than CDVs. Both histograms for AP and AN show more counties centered closer to 0 μg/m^3^, mainly between −1 and 1 μg/m^3^, meaning that they exhibit lower differences than NP and NN counties. NPs (Figure S1c in Supporting Information S1) have differences ranging from 0.13 to 5.73 μg/m^3^ with a mean of 1.35 μg/m^3^ and standard deviation of 1.05 μg/m^3^. The histogram is skewed left, meaning that more NP counties have differences between 0 and 2 μg/m^3^. NNs (Figure S1d in Supporting Information S1) have differences ranging from −5.33 to −0.13 μg/m^3^ with a mean of −1.68 μg/m^3^ and standard deviation of 1.19 μg/m^3^. The histogram is skewed right, meaning that more NN counties have differences between 0 and −2 μg/m^3^.

Overall, most counties exhibit strong agreement between CDVEs and CDVs, with most disagreements concentrated near the 9 μg/m^3^ threshold and in specific geographic regions such as the Southeast, Central Valley, and parts of the Midwest. In total, sensitivity analysis shows that many counties could shift classifications with slight changes in PM_2.5_ levels, particularly non‐aligned counties reclassifying as APs or ANs. APs and ANs tend to show smaller differences between CDVE and CDV, while NPs and NNs exhibit larger discrepancies.

### 3.2 Explanatory Variables

This section explores the geography, county characteristics, and monitor and satellite‐derived data attributes of all U.S. EPA monitored counties and investigates how these factors may contribute to differences between monitor‐based CDVs and satellite‐derived CDVEs. We examine how classification outcomes (APs, ANs, NPs, and NNs) vary with county area, region type (mountain and/or desert), urban land percentage, monitor count per county, monitor‐based CDV level, spatial monitor coverage over the county, the number of satellite grid cells removed due to high uncertainty, and the distance between peak monitor and satellite‐derived values.

**Figure 2:**
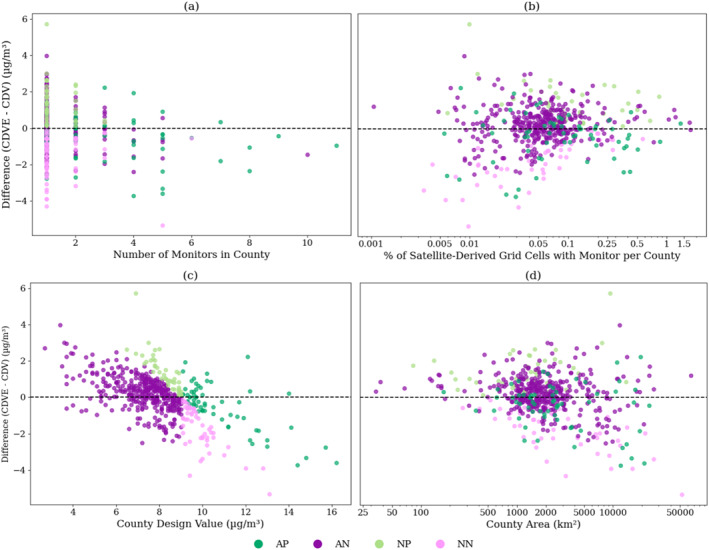
Scatter plots showing how the difference between 2023 DV period CDVs and CDVEs (CDVE–CDV) in μg/m^3^ changes with (a) number of monitors in a county, (b) the percentage of satellite‐derived data grid cells with a monitor per county, (c) the CDV, and (d) the county area in km^2^. Each plot is separated by classification: AP (green), AN (purple), NP (light green), and NN (light pink). Mean CDVE–CDV and mean x‐values for each alignment group shown in each panel are summarized in Table S1 in Supporting Information S1.

Figure 2 shows how the difference between the 2023 DV period CDVs and CDVEs changes with (a) number of monitors in a county, (b) the percentage of satellite‐derived data grid cells with a monitor per county, (c) the CDV, and (d) the county area in km^2^ by classification. Figure 2a shows how the number of monitors in a county relates to the difference between CDVEs and CDVs. The vast majority of counties (408 out of 536, or 76%) have only one monitor, with 94% (502 out of 536) having three or fewer. Very few counties have more than five monitors—only nine counties' totals exceed this threshold. Counties with one or two monitors have an absolute mean difference of 0.88 μg/m^3^ and counties with more than two monitors have an absolute mean difference of 1.08 μg/m^3^, which is 19% greater than those with one or two. This same pattern is exhibited for aligned counties (AP and AN) as well, with AP counties with one or two monitors having a 42% lower absolute mean difference and AN counties a 10% lower absolute mean difference. We have different findings when the monitors and satellite‐derived data don't align. NP counties which have one or two monitors have a 276% greater absolute mean difference than those with more than two monitors and NN counties with one or two monitors have a 10% greater absolute mean difference.

These results indicate that in counties where monitors and satellite‐derived data do not align, particularly in NP cases, a lower number of monitors may be a key contributing factor to misalignment. With only one or two monitors, localized pollution hotspots or industrial sources may be overrepresented, leading to monitor overestimation (NNs). Conversely, the monitor(s) may be located away from areas with the highest PM_2.5_ levels which satellite‐derived data may capture, resulting in underestimation (NPs). In AP counties (and AN counties to a lesser extent), higher monitor counts may have led to much greater absolute mean differences because additional monitors are often sited in areas of concern which increases the likelihood of capturing localized pollution extremes. Since the monitor‐based CDV reflects the maximum value across all monitors in a county, this can elevate the CDV beyond what is reflected in satellite‐derived estimates, which represent a spatial percentile (90th) across the county. This mismatch in aggregation methods can widen the difference even when both sources agree on the county's classification. This effect is especially evident in Figure 2a, where nearly all differences at higher monitor counts are negative, indicating that monitor‐based CDVs consistently exceed satellite‐derived CDVEs.

Figure 2b shows how the percentage of satellite‐derived grid cells with monitors in each county compares to the difference between CDVEs and CDVs. Because the resolution of the satellite‐derived data is so fine (1 km × 1 km), the percent of grid cells in a county containing a monitor is very small—even in counties with multiple monitors. Most counties (79%) have monitors in less than 0.1% of their grid cells, reflecting the sparsity of ground‐based monitoring relative to the high spatial density of the satellite‐derived data grid. In counties where less than 0.05% of grid cells contain monitors, CDVE–CDV differences span a wide range (−5.33 to 5.73 μg/m^3^), reflecting substantial variability. As monitor coverage increases to 0.05%–0.1%, the absolute mean difference decreases by 39%, suggesting improved agreement between satellite‐derived and monitor values. However, moving from the 0.05%–0.1% range to >0.1% coverage results in a 13% increase in the absolute mean difference relative to the 0.05%–0.1% group, indicating that beyond a certain threshold, additional monitor coverage may reintroduce discrepancies. This increase is driven by seven counties with large CDVE–CDV absolute differences (>2 μg/m^3^), primarily caused by monitor representativeness rather than systematic satellite bias. In these cases, the maximum monitor was either located within localized PM_2.5_ hotspots (yielding higher CDVs) or in lower‐concentration areas relative to the satellite‐derived grid distribution of PM_2.5_ (yielding higher CDVEs), or the monitor was located in higher‐concentration areas according to satellite‐derived grids but recorded lower concentrations than surrounding grid cells (higher CDVEs). When these seven counties are removed, the mean absolute difference for the >0.1% coverage group becomes nearly identical to that of the 0.05%–0.1% coverage group, indicating that the small 13% increase is driven by these seven high‐difference counties. These results persist when broken down by classification as well, with the exception of AN counties, which show a consistent decline in absolute differences as spatial coverage increases.

Figure 2c shows how the monitor‐based CDV relates to the difference between CDVEs and CDVs. The CDVE‐CDV difference is highest in magnitude at both low and high CDV levels, with a Pearson *r* of −0.60 indicating that differences become more negative as CDV levels increase. Since we noticed a pattern that counties with lower CDVs (<7 μg/m^3^) tend to have positive differences, those with higher CDVs (>10 μg/m^3^) often show negative differences, and the 7–10 μg/m^3^ range corresponds to the lowest overall differences, we grouped counties into three CDV bins (<7, 7–10, and >10 μg/m^3^) to further examine these patterns by classification.

In the <7 μg/m^3^ bin, all counties are ANs (32% of all ANs) or NPs (7% of all NPs). These counties usually show positive differences, meaning satellite‐derived values exceed monitor‐based values: the mean difference is 3.59 μg/m^3^ for NPs and 0.82 μg/m^3^ for ANs, with 100% of NPs and 82% of ANs having positive differences. This suggests that in low‐CDV counties, the county‐wide 90th percentile aggregated satellite data tends to be higher than the county‐wide maximum of PM_2.5_‐monitored locations. In the 7–10 μg/m^3^ range, where the most counties fall, all classifications are represented, with 93% of NPs, 63% of NNs, 68% of ANs, and 46% of APs falling within this range. APs and ANs have mean differences closer to zero (0.20 and –0.03 μg/m^3^, respectively), and classifications are more evenly split between positive and negative differences, especially ANs (53% positive vs. 47% negative). NPs have a mean difference of 1.19 μg/m^3^ and NNs have a mean difference of −1.13 μg/m^3^. These lower differences reinforce that agreement is strongest in the mid‐CDV range. In the >10 μg/m^3^ bin, all counties are APs (54% of all APs) and NNs (37% of all NNs). The mean difference is −1.06 μg/m^3^ for APs and −2.64 μg/m^3^ for NNs, with 79% of APs and 100% of NNs having negative differences. These results suggest that in high‐CDV counties, the county‐wide maximum of PM_2.5_‐monitored locations tends to exceed the county‐wide 90th percentile aggregated satellite‐derived values.

Figure 2d shows how the difference changes with the size of a county. All EPA monitored counties have a mean county area of 3,453.90 km^2^, a standard deviation of 5,349.64 km^2^, a median of 1,812.49 km^2^, and a range from 33.54 km^2^ to 64,681.95 km^2^. Most counties are relatively small in area, with the majority of points clustered below 5,000 km^2^ and the 90th percentile extending to only ∼8,130 km^2^ while the maximum is significantly higher at ∼64,682 km^2^. Following the clustering of points below 5,000 km^2^ in Figure 2d, we define small counties as those less than or equal to 5,000 km^2^ and large counties as those greater than 5,000 km^2^. With these classifications, we find that larger counties tend to have a 91% greater absolute difference between CDVEs and CDVs than smaller counties, indicating that the size of a county may be contributing to differences between monitors and satellite‐derived data. This pattern is consistent with expectations, as satellite‐derived data in smaller counties is aggregated over a limited spatial extent, making it more likely to capture conditions similar to those observed at ground monitors. In contrast, larger counties encompass more heterogeneous terrain and emissions sources, increasing the potential for discrepancies. Both aligned and non‐aligned counties also exhibit higher absolute differences in larger counties, with aligned counties having a 74% higher absolute mean difference than smaller counties and non‐aligned counties a 126% higher absolute mean difference. Differences are especially large for larger counties where the monitors and satellite don't align (NP and NN). When broken down by classification, large AP counties have a mean difference of −0.74 μg/m^3^, while small AP counties have a mean difference of −0.391 μg/m^3^; large NP counties a mean difference of 3.20 μg/m^3^ and small NP counties a mean difference of 1.21 μg/m^3^; and large NN counties a mean difference of −2.76 μg/m^3^ and small NN counties a mean difference of −1.29 μg/m^3^. AN counties exhibit the opposite, with large counties having a mean difference of −0.11 μg/m^3^ and small counties having a mean difference of 0.31 μg/m^3^.

**Figure 3:**
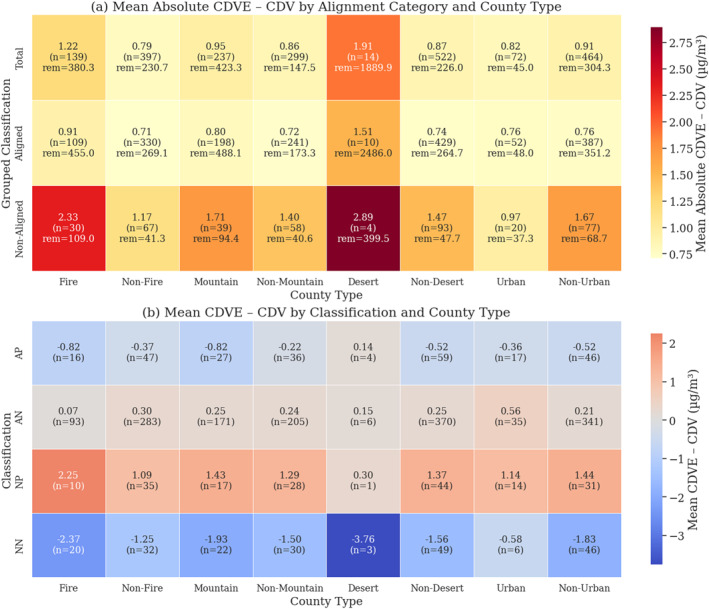
A heatmap showing CDVE–CDV differences by classification outcome and county type. (a) Mean absolute differences between CDVE and CDV for grouped classification outcomes (Aligned: AP and AN; Non‐Aligned: NP and NN; Total: all counties) and across eight county types (Fire, Non‐Fire, Mountain, Non‐Mountain, Desert, Non‐Desert, and Urban and Non‐Urban). (b) Mean differences between CDVE and CDV are shown across the four classification categories (AP, AN, NP, NN) and the same county types as (a), where cool colors indicate a higher CDV than CDVE, while warm colors indicate a higher CDVE than CDV. Cell annotations include mean values, sample sizes (n), and the mean number of satellite‐derived uncertain grid cells removed (rem; panel a only).

Figure 3 shows how the difference between 2023 DV period CDVs and CDVEs (CDVE–CDV) changes with varying county or region types, exploring wildfires, mountains, deserts, and urban areas by aligned, non‐aligned, and all counties (Figure 3a) and by classification (Figure 3b). EPA monitored counties that have had one or more wildfires from 2021 to 2023 (*N* = 139) usually have higher differences than counties that have had no wildfires within that period (*N* = 397). Overall, counties with wildfires have a 54% higher absolute mean difference, 72% higher standard deviation, and a 63% higher range than counties that didn't have a wildfire occurrence. Both aligned counties and non‐aligned counties had greater absolute differences with wildfires, than without, with aligned counties having a 28% higher absolute mean difference and non‐aligned counties being 99% higher. This means that wildfires contribute to differences between the monitors and satellite‐derived data, especially within counties where they don't agree on if the county is above or below the threshold (NP and NN). When examined by classification, 16 of the 63 (25%) AP counties had wildfires between 2021 and 2023, 93 of the 376 (25%) AN counties had wildfires, 10 out of the 45 (22%) NP counties had wildfires, and 20 out of the 52 (38%) NN counties had wildfires. Of the counties that had wildfires, the acres burned per AP county ranged from 594 acres to 1,088,163 acres, with an average of 183,115.9 acres; the acres burned per AN county ranged from 503 acres to 352,046 acres, with an average of 18,812.3 acres; NP counties had a range of 642 acres to 1,101,349 acres and average of 228,868.2 acres; and NN counties had a range of 526 acres–543,458 acres and average of 105,980.9 acres (Figure S2 in Supporting Information S1). The AN county average of acres burned is much lower (82%–92% lower) than that for the NP, NN, and AP counties, indicating that wildfires could be one of the main reasons as to why a county is greater than the NAAQS threshold.

Counties that had a wildfire occur between 2021 and 2023 (the DV period), tend to have higher mean differences than counties that didn't have wildfires within counties classified by monitors and/or satellite‐derived data (NN, NP, and AP) as being greater than the NAAQS threshold. NP wildfire counties have a mean difference of −2.37 μg/m^3^, while non‐fire counties a mean difference of −1.25 μg/m^3^; NN wildfire counties a mean difference of 2.25 μg/m^3^ and non‐wildfire counties have a mean difference of 1.09 μg/m^3^; and AP wildfire counties a mean difference of −0.82 μg/m^3^ and non‐wildfire counties a mean difference of −0.37 μg/m^3^. AN counties observed the opposite, with wildfire counties (*N* = 93) having a mean difference of 0.07 μg/m^3^ and non‐wildfire counties (*N* = 283) having a mean difference of 0.30 μg/m^3^. This further reinforces the idea that wildfires could have a significant effect on whether or not a county is classified as being above the NAAQS threshold, and could be a factor in the differences seen between CDVs and CDVEs in non‐aligned counties.

Counties that are classified as being within a mountainous region (*N* = 237) usually have higher differences than counties that are not within a mountainous region (*N* = 299). Overall, mountain regions have a 10% higher absolute mean difference, a 13% higher standard deviation, and a 6% lower range than counties that are not in mountainous regions. Both aligned counties and non‐aligned counties had greater absolute differences with mountains, than without, with aligned counties having an 11% higher absolute mean difference and non‐aligned counties being 22% higher. This means that mountains may contribute to differences between monitors and satellite‐derived data, especially within counties where they don't agree on if the county is above or below the threshold (NP and NN). When broken down by classification, counties within mountainous regions still appear to have higher mean differences between CDVEs and CDVs than counties within non‐mountainous regions. AP mountain counties (*N* = 27) have a mean difference of −0.82 μg/m^3^, while AP non‐mountain counties (*N* = 36) have a mean difference of −0.22 μg/m^3^; AN mountain counties (*N* = 171) a mean difference of 0.25 μg/m^3^ and AN non‐mountain counties (*N* = 205) a mean difference of 0.24 μg/m^3^; NP mountain counties (*N* = 17) a mean difference of 1.43 μg/m^3^ and NP non‐mountain counties (*N* = 28) a mean difference of 1.29 μg/m^3^; and NN mountain counties (*N* = 22) a mean difference of −1.93 μg/m^3^ and NN non‐mountain counties (*N* = 30) a mean difference of −1.50 μg/m^3^. There is a very small difference between AN mountain and non‐mountain counties (one hundredth) compared to the other classifications, so mountain regions may be contributing to both differences between monitors and satellite‐derived data, as well as whether or not a county is above the NAAQS threshold.

Counties that are classified as being within a desert (*N* = 14) usually have higher differences than counties that are not within a desert (*N* = 522), but not when broken down by classification. Overall, counties within deserts have a 120% higher absolute mean difference, a 103% higher standard deviation, and a 12% lower range than counties not in a desert. Both aligned counties and non‐aligned counties had greater absolute differences when they were within a desert, than when they weren't, with aligned counties having a 104% higher absolute mean difference and non‐aligned counties being 97% higher. This means that deserts may contribute to differences between monitors and satellite‐derived data in all types of counties. When broken down by classification, counties within deserts appear to have lower differences between CDVEs and CDVs than counties not in a desert. The mean difference for desert counties is less than the mean difference for non‐desert counties for NP and AP counties, is greater for NN counties, and is nearly negligible for AN counties. AP desert counties (*N* = 4) have a mean difference of 0.14 μg/m^3^, while AP non‐desert counties (*N* = 59) have a mean difference of −0.52 μg/m^3^; AN desert counties (*N* = 6) a mean difference of 0.15 μg/m^3^ and AN non‐desert counties (*N* = 370) a mean difference of 0.25 μg/m^3^; NP desert counties (*N* = 1) a mean difference of 0.30 μg/m^3^ and NP non‐desert counties (*N* = 44) a mean difference of 1.37 μg/m^3^; and NN desert counties (*N* = 3) a mean difference of −3.76 μg/m^3^ and NN non‐desert counties (*N* = 49) a mean difference of −1.56 μg/m^3^. There are very few desert counties (*N* = 14), so the statistics of small numbers may allow for a greater likelihood that a few extreme cases might affect the averages. Desert areas also tend to have the highest mean number of uncertain satellite‐derived GL grids removed in our data filtering step due to high uncertainty than any other category. We will further discuss the uncertain grids removed for all categories later in this section.

Counties that are classified as having a large percentage of urban area (≥50% urban area; *N* = 72) usually have lower differences than counties that have a lower percentage of urban area (<50% urban area; *N* = 464). Overall, more urbanized counties have a 10% lower absolute mean difference, a 28% lower standard deviation, and a 58% lower range than less urbanized counties. Aligned counties had no absolute difference and non‐aligned counties had a greater absolute difference when they were outside of an urban area, than within an urban area, with non‐aligned counties having a 72% higher absolute mean difference. This means that non‐urban areas may contribute to differences between monitors and satellite‐derived data within counties where they don't agree on if the county is above or below the threshold (NP and NN). When broken down by classification, we find that AP, NP, and NN counties tend to have a smaller mean difference for urban counties than non‐urban counties. AP urban counties (*N* = 17) have a mean difference of −0.36 μg/m^3^, while non‐urban AP counties (*N* = 46) have a mean difference of −0.52 μg/m^3^; NP urban counties (*N* = 14) a mean difference of 1.14 μg/m^3^ and non‐urban NP counties a mean difference of 1.44 μg/m^3^ (*N* = 31); and NN urban counties (*N* = 6) have a mean difference of −0.58 μg/m^3^ and non‐urban NN counties (*N* = 46) a mean difference of −1.83 μg/m^3^; and. This pattern was the opposite for AN counties, with urban counties (*N* = 35) having a mean difference of 0.56 μg/m^3^ and non‐urban counties (*N* = 341) a mean difference of 0.21 μg/m^3^.

We also explore how the percentage of urban land within a county affects classifications as well as differences between CDVs and CDVEs. Although no strong linear relationship is observed between urban land percentage and the CDVE–CDV difference, the variability in these differences tends to be lower in more urbanized counties, suggesting somewhat improved agreement between satellite‐derived estimates and monitor values in those areas (Figure S3 in Supporting Information S1). However, we find distinct classification patterns emerging by level of urbanization (Figure S3 in Supporting Information S1). APs and NPs tend to occur in more urbanized counties, with mean urban land percentages of 37% and 41%, respectively (medians of 32% and 29%). In contrast, NNs and ANs are more concentrated in less urban counties, with much lower mean and median urban land percentages (mean of 16% and median of 3% for NPs; mean of 17% and median of 9% for ANs). This suggests that in more urban areas, satellite‐derived estimates are more likely to align with or exceed monitor‐based values, while in less urban areas, monitors are more likely to report higher values than satellites. Monitors in non‐urban areas are often sited for purposes other than tracking population exposure—such as monitoring emissions from power plants or industrial facilities. When monitors are placed close to these high‐emitting point sources, they may record elevated PM_2.5_ concentrations that do not reflect the broader surrounding area. Our approach of taking the 90th percentile of satellite‐derived values across the entire county introduces additional spatial smoothing, potentially diluting these localized peaks, while monitor‐based CDVs reflect the single highest monitor design value within a county. This methodological difference may further contribute to cases where monitor values exceed satellite‐derived estimates in less urban areas.

Figure 3a also shows the mean number of high‐uncertainty satellite‐derived grid cells removed from each county due to satellite data quality filtering for all, aligned, and non‐aligned counties (shown in the “rem” label of panel a). For all counties, deserts have the highest mean number of uncertain grids removed (rem = 1889.9), followed by mountains (rem = 423.3), then wildfires (rem = 380.3), and finally non‐urban areas (rem = 304.3). This pattern is the same for aligned counties, but for non‐aligned counties mountains and wildfires are switched. The categories that exhibit the highest absolute mean differences (wildfire, mountain, desert, non‐urban) always have more uncertain grids removed than categories with the lowest differences (non‐wildfire, non‐mountain, non‐desert, urban). There is also a contradictory pattern of aligned counties having many more uncertain grids removed than non‐aligned counties in these geographic categories, indicating that aligned counties that exhibit the characteristics that usually drive differences between CDVEs and CDVs may actually agree because more uncertain grid cells from the satellite‐derived data have been removed. Summing the mean number of uncertain grids removed across all categories, aligned counties have 4,535.4 mean uncertain grids removed, which is 441% higher than the number in non‐aligned counties (838.5). Since sometimes uncertain grid removals drive higher differences, and other times they drive lower differences, we exclude this from further analysis.

We also explore how the distance between the highest monitor and the highest satellite‐derived CDVE grid cell within a county relates to CDVE–CDV differences across classifications (Figure S4 in Supporting Information S1). APs exhibit a bell‐shaped median trend where differences become more negative through the 15–20 km bin (median: −0.55 μg/m^3^), then begin to shift more positive, with a final median of −0.02 μg/m^3^ in the 50+ km bin. APs have the highest overall mean distance of 34.23 km. ANs also show a subtle bell curve, with medians becoming more positive through the 10–15 km bin (median: 0.40 μg/m^3^), then gradually declining to 0.03 μg/m^3^. ANs have the widest overall range of distances, ranging from 0.14 to 205.42 km. NNs become increasingly negative with distance, with the median falling from −0.60 μg/m^3^ in the 0–5 km bin to −2.17 μg/m^3^ in the 50+ km bin, except for a brief increase to −0.85 μg/m^3^ in the 20–30 km bin. NPs, unlike NNs, show no clear trend—median differences fluctuate across distance bins, increasing and decreasing multiple times. They also have the lowest overall mean distance of 21.80 km from the highest monitor to the highest grid. These results suggest that spatial separation between monitors and satellite‐derived peaks may contribute to the differences in NPs, which show increasing disagreement with distance, but not other classifications.

Overall, these results demonstrate that spatial and environmental context, particularly county size, wildfire activity, mountains, deserts, and urbanization, may play a significant role in shaping the differences between satellite‐derived and monitor‐based air quality metrics. Attributes related to the monitoring network also may have a large effect on differences—specifically monitor count, CDV level, and spatial coverage of monitors—are the most explanatory for CDVE–CDV differences. Monitor‐to‐grid distance is also informative for NP counties, where differences increase with distance, though this relationship is non‐existent for other classifications, so this is excluded from further analysis, along with uncertain grid removals which also had inconclusive results.

### 3.3 Error‐Risk Assessment

This section assesses the “error‐risk” or the risk of high differences between CDVEs and CDVs within counties using the variables we found to be explanatory for differences in Section 3.2. The following eight characteristics of counties are explanatory for creating greater differences between CDVEs and CDVs, or contribute to a higher “error‐risk” using the mean absolute difference framework: counties with less than or equal to two monitors, counties with low CDV (<7 μg/m^3^) or high CDV (>10 μg/m^3^), counties that have a percent of satellite‐derived grids with monitors less than 0.05% or greater than 0.1%, counties that have an area greater than 5,000 km^2^, counties that had a wildfire occurrence(s) from 2021 to 2023, counties that are within a mountainous area, counties that are within a desert area, and counties that have a level of urbanization less than 50% (low urbanization). We call these characteristics “risk factors” from now on.

To further validate these risk factors, we performed a Mann‐Whitney U test comparing the distributions of absolute mean differences between high‐risk and low‐risk categories for the non‐aligned counties (NP and NN). After applying a conservative Bonferroni correction for 10 comparisons, high or low CDV, large county size, and wildfire occurrence were statistically significant (Table S2 in Supporting Information S1). Although not all risk factors identified through mean absolute differences were significant under the Mann–Whitney U test, the overall pattern was consistent—lower *p*‐values generally corresponded to larger absolute mean differences (except for desert counties where small sample size is an issue). This alignment supports our interpretation that the most statistically significant variables are also those exerting the strongest influence on CDVE–CDV discrepancies. Accordingly, our subsequent error‐risk assessment later in this section integrates these relationships by weighting risk factors according to the magnitude of their observed impact, which inherently reflects both their effect size from their absolute mean difference, and statistical significance.

**Figure 4:**
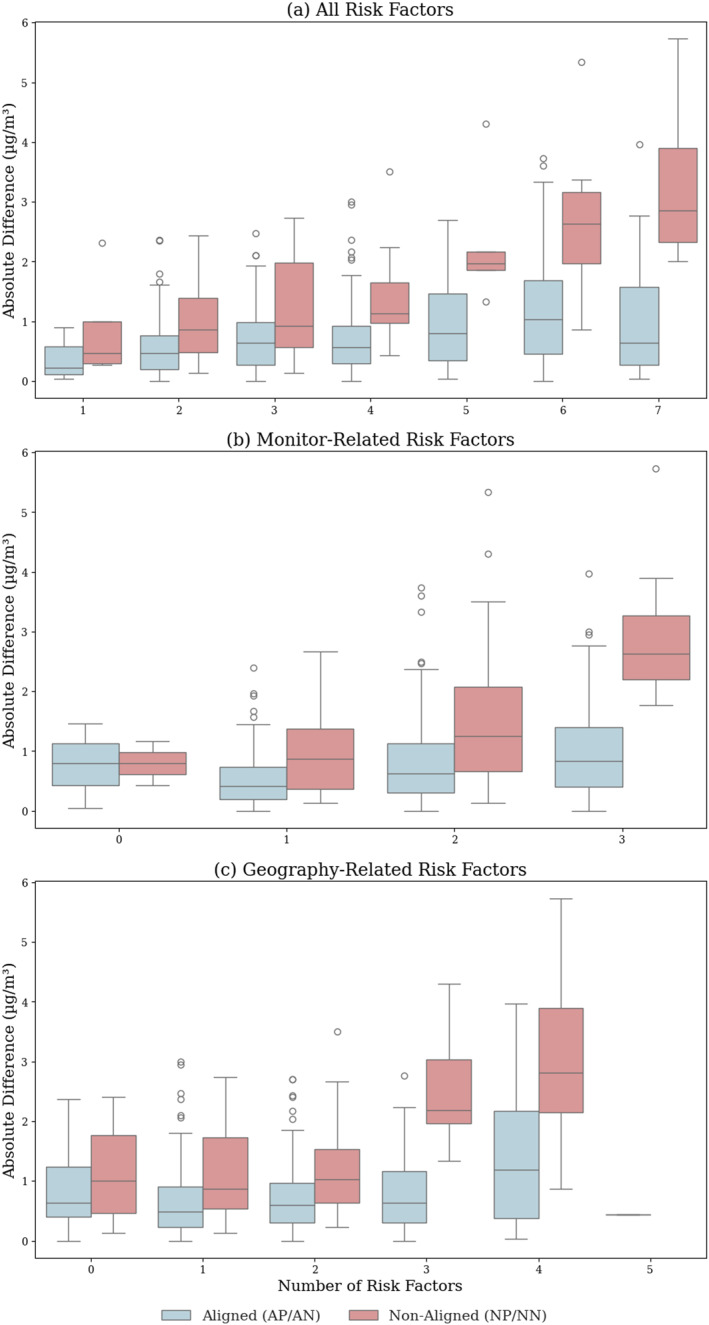
Number of risk factors per county and how they affect absolute differences between CDVEs and CDVs for aligned (light blue; AP/AN) and non‐aligned (coral; NP/NN) counties for (a) all eight risk factors, (b) risk factors related to monitors (monitor count, monitor coverage percentage, CDV), and (c) risk factors related to geography and county characteristics (county size, wildfires, mountains, deserts, and urbanization). The boxes represent the interquartile range (IQR = 75th percentile‐25th percentile), the whiskers extend to the most extreme data points within 1.5x IQR of Q1 and Q3, and any data points beyond this range are plotted individually as white circles (outliers). The middle bars are the medians. Note: all counties had at least one identified risk factor and none exhibited all eight, therefore there are no counties with zero or eight total risk factors in panel (a). This is not the case when only monitor‐related or geography‐related factors are considered.

Figure 4 shows how the absolute difference between CDVEs and CDVs varies with the number of risk factors present for each county, separated by classification group, where NP and NN counties are grouped together (non‐aligned) and AP and AN counties are grouped together (aligned). Figure 4a shows how absolute differences change with all eight risk factors. Counties with non‐alignment (in coral) always show higher absolute median differences than those with alignment (in light blue), particularly as the number of risk factors increases. This trend is most pronounced between 4 and 5 risk factors, where the spread and median of the non‐aligned group increase significantly relative to the aligned group. Notably, the median difference among non‐aligned counties continues to rise with each additional risk factor, increasing from 0.47 μg/m^3^ for one risk factor to 2.85 μg/m^3^ for seven risk factors (a 506% increase), suggesting that the accumulation of multiple contributing factors can greatly amplify differences between CDVEs and CDVs. The median difference among alignment counties usually increases with increasing risk factors, except going from 3 to 4 factors and 6 to 7 factors, but to a less extent, with the lowest absolute median difference at one risk factor being 0.22 μg/m^3^ and the highest at six risk factors being 1.03 μg/m^3^ for an increase of 368%. Overall, the results support the idea that the presence of multiple risk factors interacting increases the likelihood and magnitude of differences between satellite‐derived CDVEs and monitor‐based CDVs in non‐aligned counties.

Figure 4b shows how absolute differences change with the three risk factors related to monitors: the number of monitors per county, the monitor coverage percentage, and the CDV. Similar patterns emerge for both non‐aligned and aligned counties, with the non‐aligned group still having much higher median absolute differences than the aligned group, except for at 0 risk factors where there is a very small difference between the two. Non‐aligned counties consistently increase with increasing number of risk factors, increasing by 229% from zero risk factors (0.80 μg/m^3^) to three risk factors (2.63 μg/m^3^). Aligned counties again see nearly the same pattern, except they decrease from zero risk factors to one risk factor and then increase up to a median absolute difference of 0.83 μg/m^3^ at three risk factors. Figure 4c shows how absolute differences change with the five risk factors related to geography and county characteristics: large county size, the presence of wildfires, mountains, deserts, and low urbanization. The only difference here is that differences increase with increasing number of risk factors except from zero to one risk factor, otherwise there is a 224% increase from one to four risk factors (there are no non‐aligned counties with five risk factors). Aligned counties show the same pattern as in Figure 4b, with differences decreasing from zero to one risk factors and then increasing up to a median absolute difference of 1.18 μg/m^3^ at four risk factors (there is only one county with five risk factors so we ignore this).

Since the absolute difference increases with increasing number of risk factors in each county and with statistical significance for non‐aligned counties, we compute error‐risk scores by weighing each risk factor by how much it contributes to differences between CDVs and CDVEs in non‐aligned counties. See methods Section 2.4 for more details on this calculation. Table 1 shows the high error‐risk group and low error‐risk group definitions for all eight identified risk factors and their corresponding weights used to determine risk scores for EPA monitored counties (all risk factors) and all U.S. counties (non‐monitor related factors). When all risk factors are considered, CDV has the highest weight (therefore is contributing to the most risk), followed by county size and desert regions. When only non‐monitor related risk factors are considered, county size has the highest weight, followed by desert regions and wildfire occurrences.

**Table 1:** High Error‐Risk Group and Low Error‐Risk Group Definitions for All Eight Identified Risk Factors and Their Corresponding Weights Used to Determine Risk Scores for EPA Monitored Counties (All Risk Factors) and All U.S. Counties (Non‐Monitor Related Factors)

Variable	High error‐risk factors	Low error‐risk factors	Weights for all risk factors	Weights for non‐monitor risk factors
Monitor Count	≤2 monitors	>2 monitors	0.040	n/a
CDV	CDV < 7 μg/m^3^ or CDV > 10 μg/m^3^	CDV between 7 and 10 μg/m^3^	0.210	n/a
Monitor Coverage (% Satellite‐Derived grids w/monitors)	monitor coverage <0.05% or >0.1%	monitor coverage between 0.05% and 0.1%	0.070	n/a
Fire Region	Counties intersecting wildfire areas (2021–2023)	Counties without wildfire occurrence	0.153	0.225
Mountain Region	Counties intersecting mountain areas	Counties not intersecting mountain areas	0.041	0.060
Desert Region	Counties intersecting desert areas	Counties not intersecting desert areas	0.186	0.274
Urbanization	Counties <50% urbanized (Non‐Urban)	Counties ≥50% urbanized (Urban)	0.092	0.136
County Size	Large (>5,000 km^2^)	Small (≤5,000 km^2^)	0.208	0.305

*Note*. County size, wildfire regions, mountain regions, desert regions, urbanization (Non‐Monitor Related Factors).

**Figure 5:**
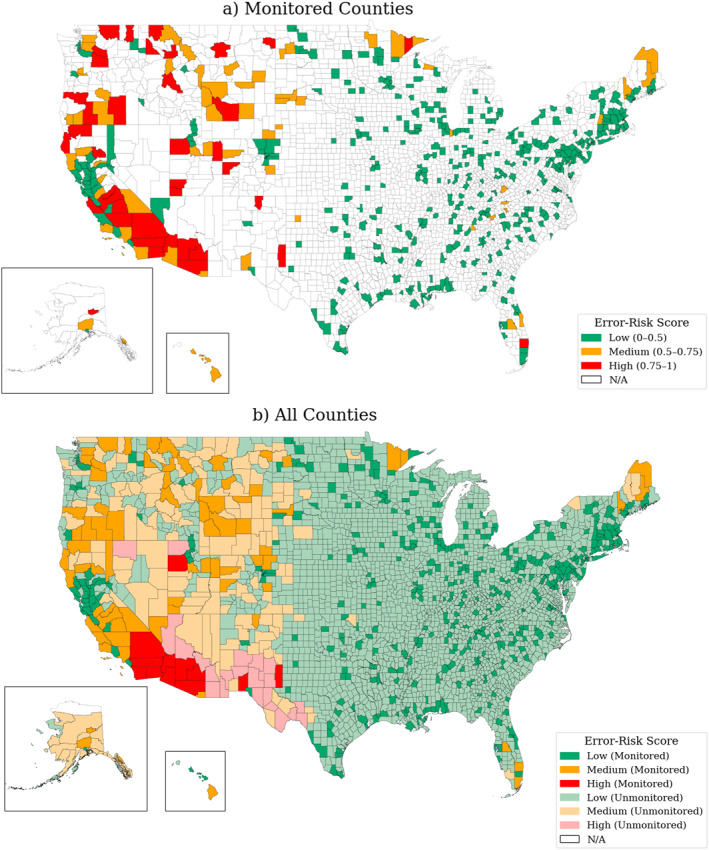
Spatial representation of the error‐risk assessment showing (a) monitored counties where error‐risk scores are computed using all eight explanatory variables, and (b) both monitored (darker colors) and unmonitored counties (lighter colors) where error‐risk scores are computed without the three monitor‐related variables. Low error‐risk counties are in green and have scores ranging from 0 to 0.5, medium error‐risk scores are in orange and have scores ranging from 0.5 to 0.75, and high error‐risk counties are in red, with scores ranging from 0.75 to 1.

Figure 5 shows the error‐risk scores for (a) EPA monitored counties, where the error‐risk score depends on all eight risk factors, and (b) all U.S. counties (except for North Slope Borough, Alaska which doesn't have satellite‐derived data), where the error‐risk score only depends on risk factors that aren't related to monitors (county size, wildfires, mountains, deserts, and urbanization). We classify counties as having low error‐risk (green) if their error‐risk scores are between 0 and 0.5, medium error‐risk (orange) if the error‐risk score is between 0.5 and 0.75, or high error‐risk (red) if the error‐risk score is between 0.75 and 1. The EPA monitored counties are shown in the darker shades and the unmonitored counties are shown in the lighter shades.

Among EPA monitored counties (Figure 5a), most low error‐risk counties are concentrated in the eastern U.S., where counties tend to be smaller, more urbanized, and have higher monitor densities. In contrast, high error‐risk counties are primarily clustered in the western U.S., especially in California, Arizona, Nevada, and parts of Montana and Wyoming. These counties are typically larger in area and are more likely to contain deserts, mountains, or recent wildfire activity, which our analysis identified as key contributors to differences between satellite‐derived CDVEs and monitor‐based CDVs. Medium error‐risk counties are scattered throughout the central and western states but are less common in the east, exhibiting risk factors such as mountains, larger county sizes, and low monitor count and coverage.

When including all counties (Figure 5b), the addition of unmonitored areas and the use of only non‐monitor‐related risk factors reveal clear spatial patterns. Large clusters of high and medium error‐risk counties are observed across the western U.S., with medium‐risk particularly in regions such as the Great Basin and the Rocky Mountains, and high‐risk in the desert areas of the Southwest. Many of these high‐risk unmonitored counties are adjacent to monitored counties with similarly high error‐risk scores, suggesting that the same geographic and environmental factors may drive elevated error potential across monitored areas from Figure 5a and unmonitored areas alike. Both monitored and unmonitored counties in the Midwest and East are overwhelmingly classified as low error‐risk, reflecting their smaller sizes, higher urbanization levels, and lack of desert or mountainous features.

Overall, these results underscore the spatial heterogeneity in error‐risk across the continental U.S. The high error‐risk clusters in western counties align with regions where our prior analysis found larger discrepancies between CDVEs and CDVs, particularly in non‐aligned counties (NP and NN) and where AP counties are located, indicating that high‐risk areas may also lead to a county being over the NAAQS threshold. Much of the ANs within the Midwest align with low‐risk areas in the Midwest. The identification of these high‐risk areas is crucial for understanding where satellite‐derived estimates may require additional scrutiny or supplemental monitoring to improve accuracy, and low‐risk areas help to identify where they may be capable of replacing monitors.

## 4 Conclusions

This study investigated associations among the differences between the county‐level EPA monitor‐based design values and satellite‐derived design value equivalents across 536 EPA monitored U.S. counties for the 2023 DV period, using the 9.0 μg/m^3^ threshold set by the 2024 PM_2.5_ NAAQS. By treating the monitor‐based CDV as the “ground truth” and the satellite‐derived CDVE as a prediction, we classified counties into aligned positives, aligned negatives, non‐aligned positives, and non‐aligned negatives. Our goal was to identify monitor‐related and geographic and county characteristics that may explain why some counties are classified differently by the satellite‐derived data and monitors, and to then perform an error‐risk assessment which identifies which counties throughout the U.S. are considered to be at risk of having large differences between CDVEs and CDVs. Here we reflect on these results and the broader implications of using sparse versus contiguous data sets in regulatory and policy decision‐making.

We identify eight variables or “risk factors” that are likely contributors to differences between CDVs and CDVEs. Counties with a low number of monitors (specifically ≤2 monitors) were frequently associated with higher differences, likely due to an insufficient number of monitors to capture county‐wide pollution patterns. Similarly, counties with low monitor coverage—specifically those with less than 0.05% of satellite grid cells containing monitors—exhibited the greatest disagreement. However, disagreement was also observed at the higher end of monitor coverage (greater than 0.1%) due to seven counties with extreme differences. In low monitor coverage cases, discrepancies were often driven by a single monitor located near a localized emission source, such as an industrial facility or traffic source, which may skew the county CDV to be much higher, making it not fully representative of the pollution levels throughout the rest of the county. The statistical significance and absolute mean differences were highest in counties with either very high (>10 μg/m^3^) or very low (<7 μg/m^3^) overall PM_2.5_ concentrations measured by monitors. This is most likely due to the monitor(s) being located near a heavily localized emission source that isn't representative of the whole county, or it being located in a lower polluting area, whereas the satellite‐derived data detects higher pollution elsewhere in the county.

Geographic and environmental features also contribute to mismatches between satellite‐derived data and monitors. Larger counties were more likely to exhibit differences between CDVs and CDVEs and were also statistically significant, reflecting the challenge of representing highly heterogeneous conditions within a single design value. Counties containing complex terrain (viz., mountains or deserts) were more likely to exhibit differences between CDVs and CDVEs. This aligns with prior research emphasizing that complex terrain introduces substantial spatial variability in meteorological conditions and pollution concentrations (Giovannini et al., 2020), making satellite‐derived products a potentially more robust solution where dense monitor networks are impractical. Counties affected by wildfires during the design value period (2021–2023) also showed larger differences and statistical significance, likely due to smoke dispersion being better captured by satellites than by fixed monitors. Counties with low urbanization levels (<50% urbanized) were more prone to differences, underscoring the uneven spatial distribution of monitors, which are more commonly sited in densely populated areas.

We also find that for counties that don't align on whether a county is above or below the 2024 NAAQS threshold (NN and NP counties), as the number of explanatory variables that a county has increases, the difference between CDVEs and CDVs also increases, with non‐aligned counties exhibiting seven risk factors having a 506% higher absolute median difference than those with one explanatory characteristic. Non‐aligned counties always exhibit a greater median absolute difference and almost always a greater spread than aligned counties (AP and AN).

Our error‐risk assessment highlights clear spatial patterns in error‐risk scores across both EPA monitored and unmonitored U.S. counties, revealing regions where satellite‐derived CDVEs may be more or less reliable proxies for monitor‐based CDVs and vice versa. Among the 536 EPA monitored counties, low error‐risk areas are concentrated in the eastern U.S., where counties tend to be smaller, more urbanized, and have denser monitoring networks. In contrast, high error‐risk counties are clustered in the western U.S., particularly in California, Arizona, Nevada, and parts of Montana and Wyoming. These high‐risk areas are characterized by large county sizes, sparse monitor coverage (except in California), complex terrain such as mountains and deserts, mostly low urbanization, and frequent wildfire activity, all of which likely contribute to substantial differences between CDVs and CDVEs. Medium error‐risk are scattered throughout the central and western states. Extending the analysis to all U.S. counties, the risk scoring framework identifies similar spatial trends. High and medium error‐risk counties again dominate the western U.S., including unmonitored regions with geographic and environmental characteristics similar to nearby monitored high‐risk counties, specifically the desert regions of the southwest. Low error‐risk counties remain concentrated in the Midwest and eastern states, where the lack of risk factors suggests that CDVEs may already provide a robust representation of surface air quality. Identifying high‐risk areas is critical for highlighting regions where satellite‐based approaches may require additional validation and adjustment, or where current monitoring isn't correctly characterizing counties. Recognizing low‐risk areas provides confidence in the application of CDVE‐based assessments and can guide efforts to prioritize resources for improving monitoring coverage in more problematic regions.

These findings underscore the importance of monitor siting in determining DV outcomes. The DV presents a clear test case for comparing sparse and contiguous data sets. Our analysis reveals that monitor location plays a significant role in determining whether a county is classified as being above or below the NAAQS threshold, especially when only a few monitors are available and spatial heterogeneity in PM_2.5_ within a county is high. Contiguous data sources like the satellite‐derived GL data set offer the advantage of spatially comprehensive coverage, allowing for the identification of pollution hotspots that may be missed by sparse, point‐based monitoring networks. However, our results also highlight the limitations of gridded data sets on the county‐level, such as their potential to miss highly localized pollution peaks when using percentile‐based metrics like the 90th percentile.

This study demonstrates that while satellite‐derived products can supplement and enhance air quality assessments, care must be taken when translating gridded estimates into policy‐relevant frameworks designed around point‐based observations. Our method of comparing CDVEs and CDVs provides a valuable tool for diagnosing gaps in monitoring networks and understanding when and where satellite‐derived products may serve as effective proxies, or where they fall short. As new satellite missions (e.g., TEMPO) and low‐cost sensor networks expand, policymakers and researchers will need to carefully consider the trade‐offs between data coverage, reliability, and relevance to regulatory standards.

This study contributes to ongoing efforts to bridge sparse and contiguous air quality data sets. By highlighting physical, environmental, and observational variables or conditions that likely drive disagreements between monitors and satellite‐derived data, we provide actionable insights for improving monitor siting strategies and for refining the integration of satellite‐derived data into regulatory and public health applications. Such approaches will be critical for ensuring comprehensive and equitable environmental observation across the U.S.

## Conflict of Interest

The authors declare no conflicts of interest relevant to this study.

## Supporting information

Supporting Information S1

## Data Availability

All data used in this study are open to the public. All Washington University Global and North American data was downloaded from the Washington University in St. Louis Atmospheric Composition Analysis Group (Hammer et al., 2023; Van Donkelaar et al., 2021; https://sites.wustl.edu/acag/datasets/surface‐pm2‐5/). The EPA county‐level design value data was downloaded from the EPA Air Quality Design Values page (US EPA, 2016; https://www.epa.gov/air‐trends/air‐quality‐design‐values). The U.S. 2020 county shapefiles were downloaded from the U.S. Census Bureau Cartographic Boundary Files page (US Census Bureau, 2025; https://www.census.gov/geographies/mapping‐files/time‐series/geo/cartographic‐boundary.2020.html#list‐tab‐1883739534). The Burned Areas Boundaries data set was downloaded from the Monitoring Trends in Burn Severity page (MTBS, 2025; https://mtbs.gov/direct‐download). The physical label areas were downloaded from the Natural Earth page (Natural Earth, 2009; https://www.naturalearthdata.com/downloads/10m‐physical‐vectors/10m‐physical‐labels/). The urban areas shapefile was downloaded from the U.S. Census Bureau (U.S. Census Bureau, 2025; https://www2.census.gov/geo/tiger/TIGER_RD18/LAYER/UAC20/). All analysis code for this study and the prior study (Holloway et al., 2026) are available in Zenodo (sjacker2, 2025; https://doi.org/10.5281/zenodo.17584000).
