# Supplementary material for: Comparison of Policy‐Relevant Air Quality Metrics Calculated With Sparse In Situ Monitoring and Contiguous Satellite‐Derived Data
Source: Geohealth. 2026 Jul 7;10(7):e2025GH001586. doi: 10.1029/2025GH001586 (PMC13339723; doi:10.1029/2025GH001586)
Supplement: Supplementary file 1 — Supporting Information S1 [file GH2-10-e2025GH001586-s001.pdf]

GeoHealth

Supporting Information for

Comparison of Policy-Relevant Air Quality Metrics Calculated with Sparse in Situ Monitoring and Contiguous Satellite-Derived Data

Summer Acker[1] Tracey Holloway[1,2*], Kevin Stewart[4], Aaron van Donkelaar[3], Randall V. Martin[3]

[1]Nelson Institute Center for Sustainability and the Global Environment, University of Wisconsin—Madison; Madison, 53705, USA.

[2] Department of Atmospheric and Oceanic Sciences, University of Wisconsin—Madison; Madison, 53705, USA.

[3] Department of Energy, Environmental & Chemical Engineering, Washington University at St. Louis; St. Louis, 63130, USA.

[4] American Lung Association, Chicago, 60601, USA.

Contents of this file

Figures S1 to S4

Tables S1 to S2

Additional Supporting Information

Captions for Figures S1 to S4

Captions for Tables S1 and S2

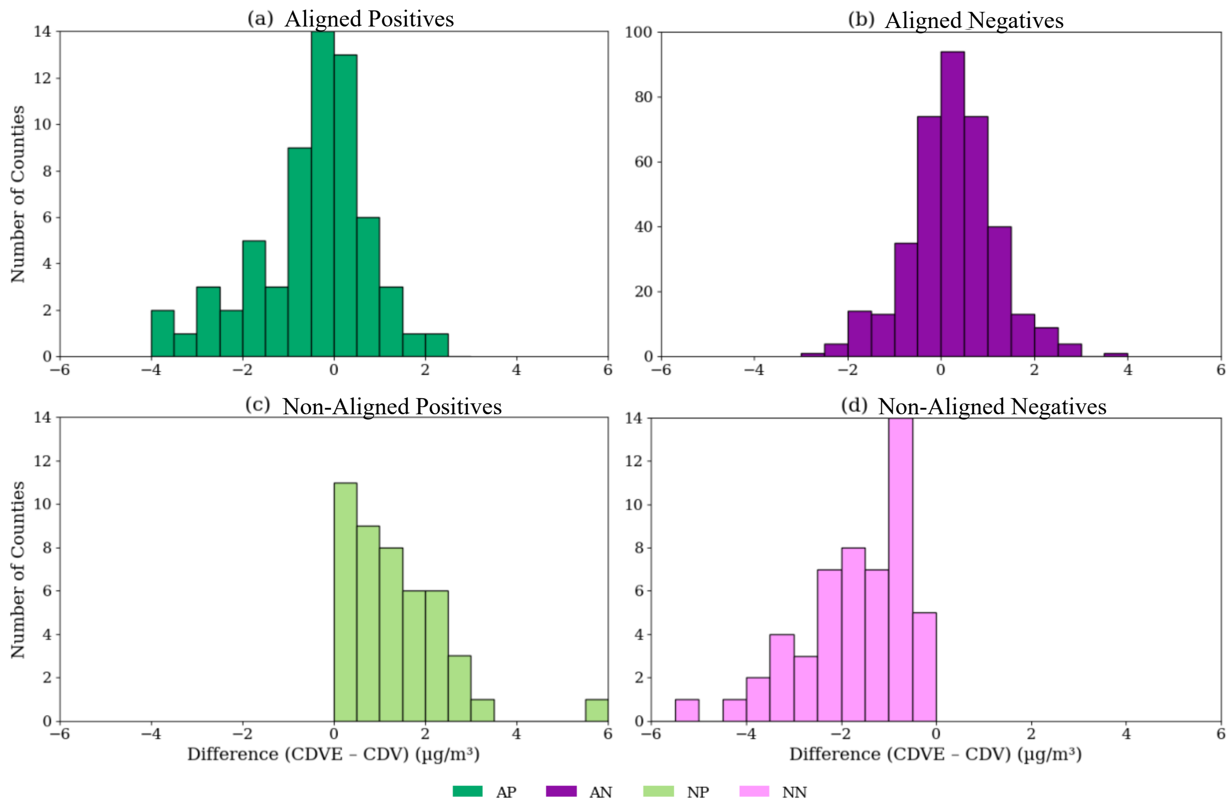

Figure S1. Distributions of the differences between CDVEs and CDVs by each classification: a) APs (green), b) ANs (purple), c) NPs (light green), and d) NNs (light pink). Please note that the AN panel has a different y-axis, extending up to 100 instead of 14 since there are so many more AN counties than the other three classifications.

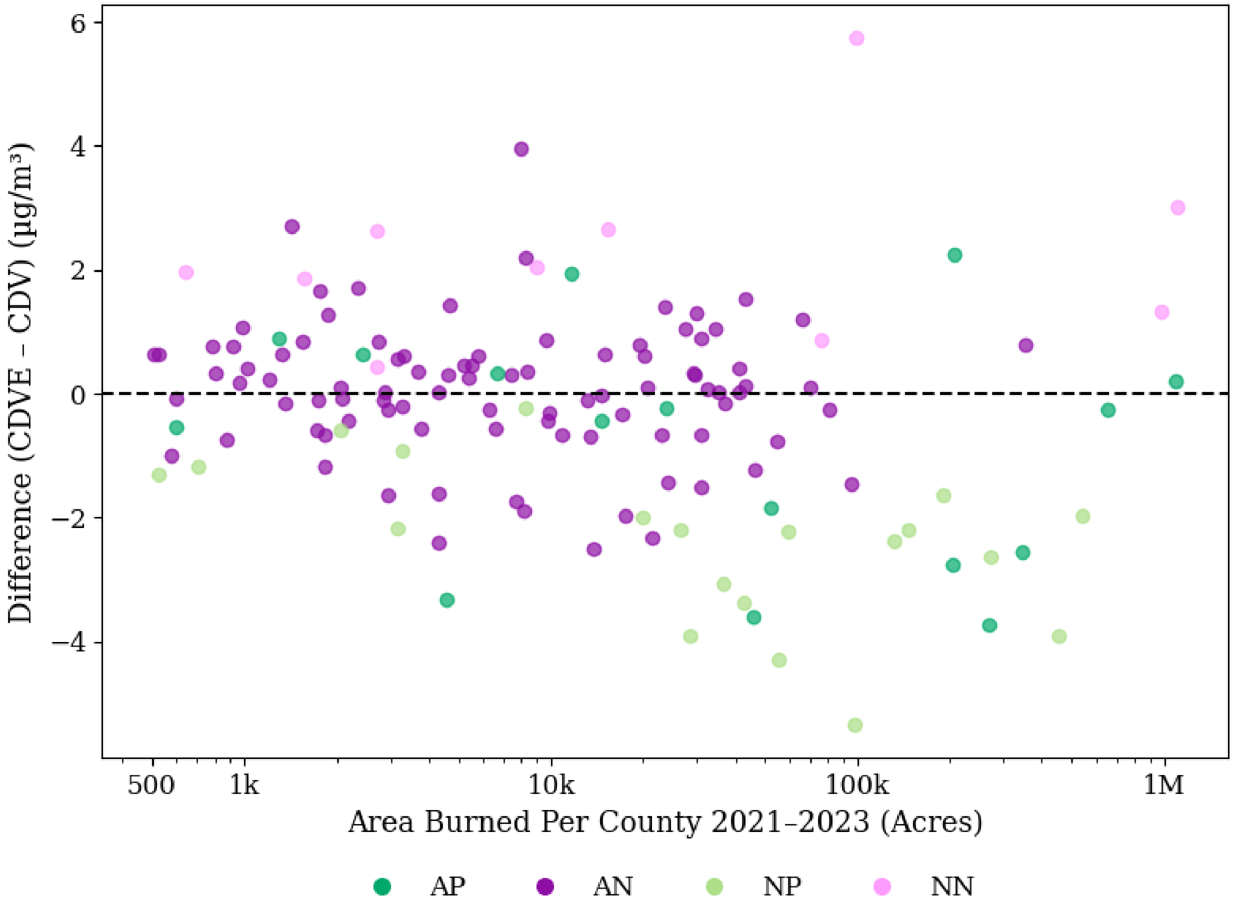

Figure S2. Scatter plot of the CDVE—CDV difference in μg/m^3 compared to the area burned by wildfires from 2021 to 2023 in acres within a EPA monitored county separated by classification: AP (green), AN (purple), NP (light green), and NN (light pink).

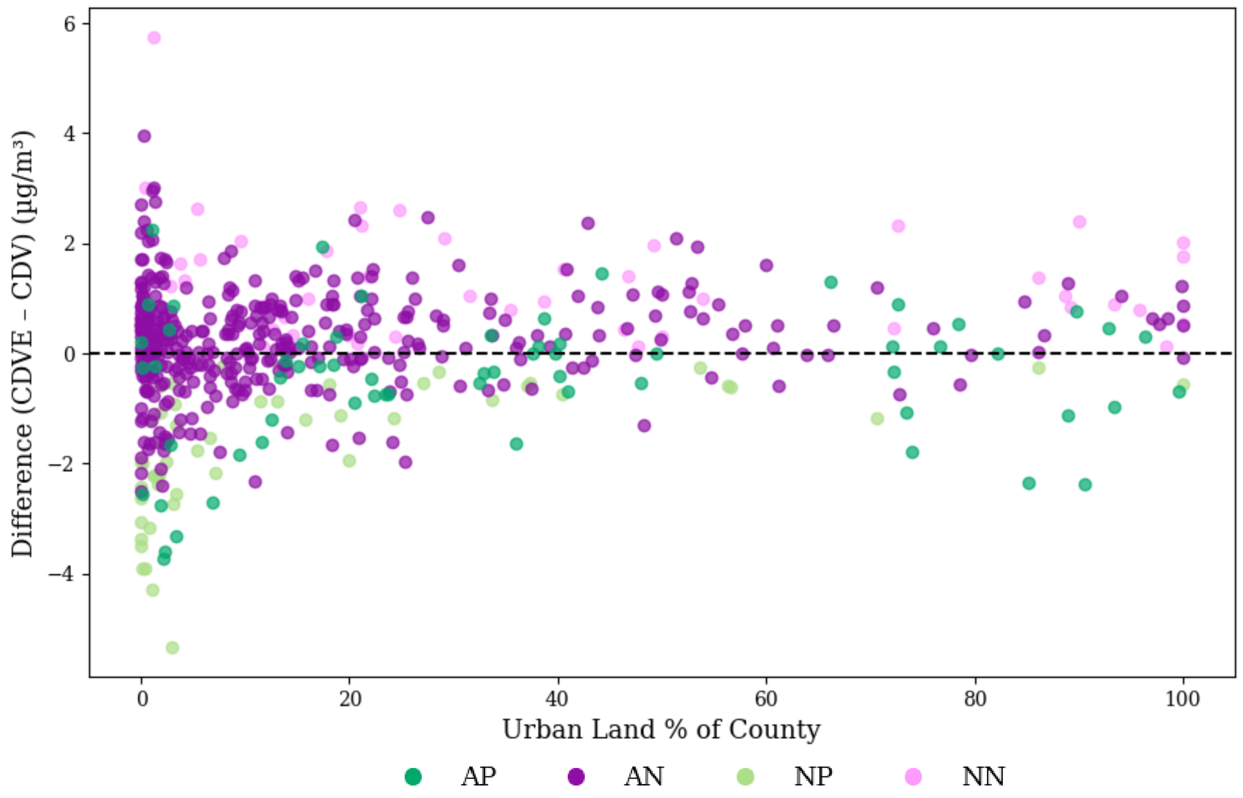

Figure S3. Scatter plot of the CDVE—CDV difference in μg/m^3 compared to the percentage of urban land within a EPA monitored county separated by classification: AP (green), AN (purple), NP (light green), and NN (light pink).

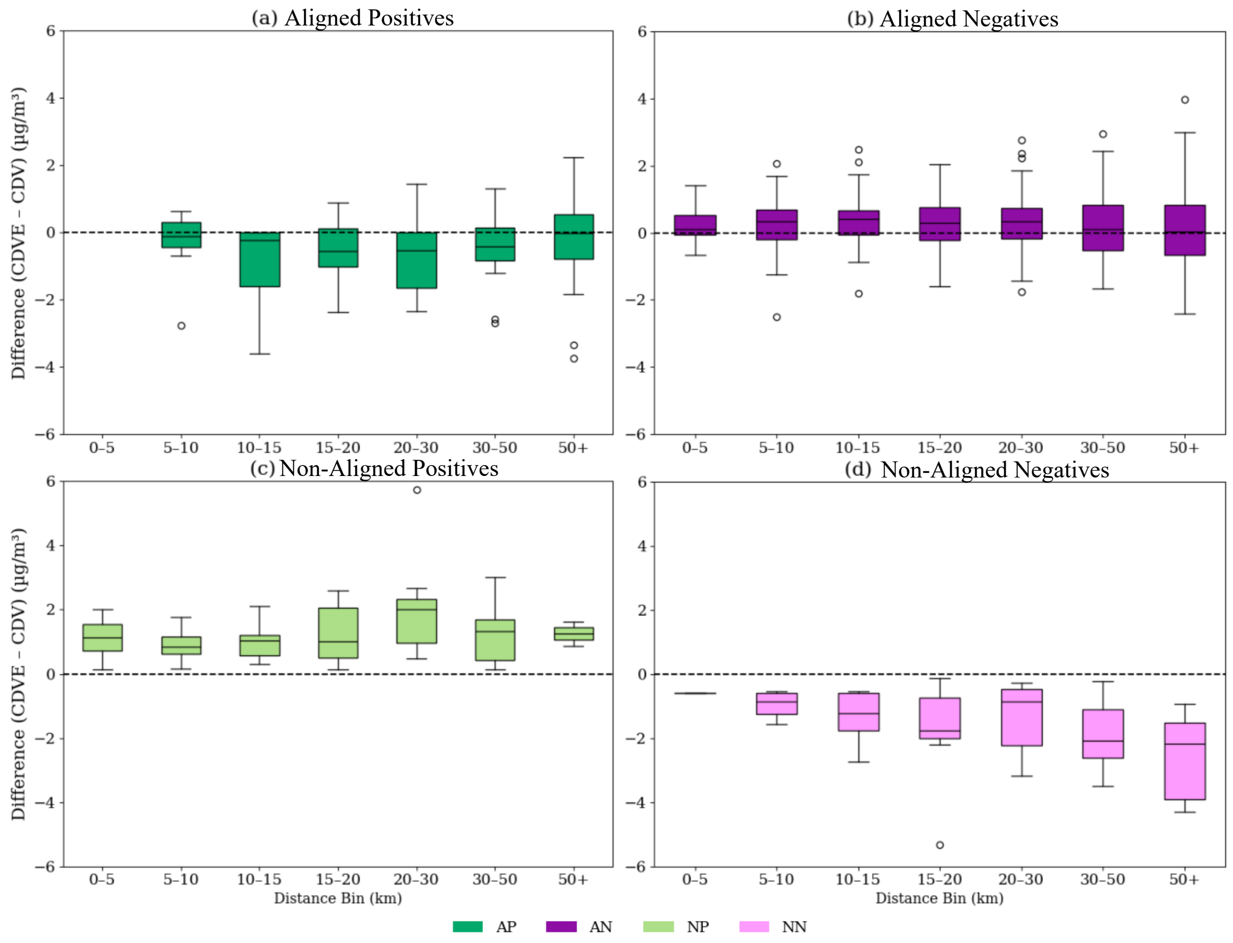

Figure S4. Boxplot of peak monitor to peak grid distance within each EPA monitored county compared to the difference between CDVs and CDVEs (CDVE – CDV) by classification: a) AP, b) AN, c) NP, and d) NN. The distance bins range from 0 km to 50 km, with an extra bin (50+ km) for all distances greater than 50 km. The boxes represent the interquartile range (IQR = 75th percentile – 25th percentile), the whiskers extend to the most extreme data points within 1.5 x IQR of Q1 and Q3, and any data points beyond this range are plotted individually as white circles (outliers). The middle bars represent the medians.

Table S1. Mean CDVE–CDV and mean x-values for each alignment group and panel of Figure 2. The mean CDVE-CDV differences will be the same for each classification across panels.

Panel A: Monitor Count		
**Classification**	**Mean Monitor Count**	**Mean CDVE-CDV Difference**
AP	3.03	-0.48
AN	1.27	0.24
NP	1.29	1.35
NN	1.52	-1.68
Panel B: Percentage of satellite-derived data grid cells with a monitor per county		
**Classification**	**Mean Monitor Coverage %**	**Mean CDVE-CDV Difference**
AP	0.16	-0.48
AN	0.09	0.24
NP	0.15	1.35
NN	0.08	-1.68
Panel C: CDV		
**Classification**	**Mean CDV**	**Mean CDVE-CDV Difference**
AP	10.76	-0.48
AN	7.22	0.24
NP	8.21	1.35
NN	9.86	-1.68
Panel D: County Area		
**Classification**	**Mean County Area**	**Mean CDVE-CDV Difference**
AP	4898	-0.48
AN	3136	0.24
NP	2004	1.35
NN	5256	-1.68

Table S2. Risk factors and their statistical significance calculated by the Mann-Whitney U test for NP and NN counties. The corresponding mean absolute differences (MAD) between high and low risk groups for NP and NN counties are also shown.

Risk Factor	High Risk	Low Risk	High MAD (μg/m^3)	Low MAD (μg/m^3)	ΔHigh – Low MAD (μg/m^3)	p-value	Bonferroni p-value
CDV	<7 or >10 μg/m^3	7 – 10 μg/m^3	2.77	1.16	1.60	1.63E-08	1.63E-07
County Size	> 5,000 km^2	≤ 5000 km^2	2.83	1.25	1.58	6.42E-06	6.42E-05
Wildfires	Fire	Non-Fire	2.33	1.17	1.17	1.62E-05	1.62E-04
Urban Category	Non-Urban	Urban	1.67	0.97	0.70	0.011	0.11
Monitor Coverage	<0.5% or >0.1%	0.5% - 0.1%	1.73	1.20	0.53	0.074	0.74
Monitor Count	≤ 2 monitors	> 2 monitors	1.55	1.24	0.31	0.080	0.80
Mountains	Mountain	Non-Mountain	1.71	1.40	0.31	0.197	1.97
Deserts	Desert	Non-Desert	2.89	1.47	1.42	0.256	2.56
